# Botanical Extract–Infused Shampoo and Hair Tonic for Hair Loss in Androgenetic Alopecia: A TREND‐Compliant, Prospective Single‐Arm Preexperimental Study

**DOI:** 10.1111/jocd.70273

**Published:** 2025-06-04

**Authors:** Dong‐Gi Choi, Woo‐Chul Shin

**Affiliations:** ^1^ Cheonho Kyunghee Korean Medicine Clinic Seoul Republic of Korea; ^2^ Department of Korean Rehabilitation Medicine Kyung Hee University, College of Korean Medicine, Kyung Hee University Korean Medicine Hospital Seoul Republic of Korea

**Keywords:** androgenic alopecia, botanical extract, global photograph, image analysis, TREND

## Abstract

**Background:**

Androgenetic alopecia (AGA) is the most prevalent form of hair loss, often leading to treatment discontinuation due to the adverse effects and perceived ineffectiveness of conventional treatments. Botanical extracts are gaining attention for their potential benefits in managing AGA with minimal side effects.

**Aims:**

To evaluate the efficacy and safety of a shampoo and hair tonic containing Asacurin100, a botanical extract blend, in improving hair loss conditions in patients with AGA.

**Methods:**

A prospective single‐group, pretest–posttest study was conducted with 35 male participants diagnosed with AGA at a Korean medicine clinic. Participants applied the shampoo once daily and the hair tonic two to three times daily for 6 months. Data were collected via standardized global scalp photographs and a custom‐designed questionnaire assessing scalp symptoms. Statistical analyses were performed to evaluate changes in hair loss area and subjective symptoms.

**Results:**

The study observed reductions in hair loss area, with a mean decrease of −27.35 ± 26.31 (*p* < 0.0001), and improvement in all scalp symptom scores. The Patient Global Impression of Change (PGIC) revealed that 80% of participants reported “much” or “very much” improvement in hair condition. No serious adverse events were reported.

**Conclusions:**

The findings suggest that botanical extract–infused shampoo and hair tonic are effective and well‐tolerated treatments for AGA, reducing hair loss and enhancing scalp health. The results warrant further investigation through larger randomized controlled trials to validate the efficacy of plant‐derived compounds for AGA.

## 1 Introduction

Androgenetic alopecia (AGA), also known as male‐pattern hair loss, predominantly affects men and is characterized by progressive, bilateral hair thinning, particularly in the frontal and central scalp regions. It is the most prevalent form of hair loss [1]. AGA results from an exaggerated response to androgens, particularly dihydrotestosterone (DHT), leading to the progressive miniaturization of hair follicles in genetically predisposed individuals. The underlying pathophysiology involves increased levels of DHT and 5‐alpha reductase type II, elevated androgen receptor density, and diminished dermal papilla growth factors [1, 2].

The prevalence of AGA varies among ethnic groups, with Asians generally exhibiting a lower prevalence than Caucasians. In South Korea, AGA affects approximately 14.1%–50% of the population, with an increasing trend consistent with global patterns [3, 4]. Despite its significant impact, many patients discontinue treatment due to concerns about the side effects of conventional therapies, perceived lack of efficacy, and poor adherence [5]. As a result, plant‐derived therapies are increasingly utilized worldwide, either as alternatives or adjuncts to conventional medicine, due to their relatively mild side effects and accessibility [2]. More than 30% of adults experiencing hair loss reportedly use nonconventional treatments, including herbal remedies [6]. While several studies suggest potential improvements in AGA with herbal therapies, the lack of controlled clinical trials and inconsistent findings have precluded evidence‐based recommendations for natural product [5]. In South Korea, traditional medicine has employed natural plant extracts for alopecia treatment, based on traditional Korean medical literature and empirical knowledge. Commonly used herbs include angelica, mulberry, sophora root, and many more. While several reviews have examined the use of botanical extracts in alopecia treatment, they consistently highlight the need for higher quality research with larger sample sizes to substantiate these treatments' efficacy [7, 8].

Our previous in vivo [9] and clinical trial [10] have demonstrated the hair loss prevention effect of a natural plant extract compound in hair loss animal models and populations. This preexperimental study aimed to evaluate the efficacy of a botanical extract–infused shampoo and hair tonic, primarily composed of herbal compounds, in alleviating AGA symptoms.

## 2 Methods

### 2.1 Study Design

This prospective single‐group, pretest–posttest study was conducted at a Korean medicine clinic in Seoul, Republic of Korea, from June 2023 to March 2024. The study followed the Transparent Reporting of Evaluations with Nonrandomized Designs (TREND) guidelines and was approved by the Public Institutional Review Board of the Ministry of Health and Welfare (Approval No. P01‐202307‐01‐010). This study was conducted in accordance with the principles of the Declaration of Helsinki.

Individuals visiting the clinic for hair loss were informed about the study through a participant information sheet, and written informed consent was obtained from those who voluntarily agreed to participate. The participants were screened based on the inclusion and exclusion criteria outlined in Table 1. Given the preliminary nature of this study, based on the previous literature [11] and considering the study duration and an anticipated drop‐out rate of 10%, approximately 35 participants were enrolled.

**TABLE 1:** Inclusion and exclusion criteria.

Inclusion criteria	1. Male and female participants aged 18–65 years diagnosed with androgenetic alopecia:
	① Diagnosed according to the Basic and Specific (BASP) classification:
	Basic type: Participants diagnosed with M1 or higher, or U1 or higher in androgenetic alopecia
	Specific type: Participants diagnosed with V1 or higher, or F1 or higher in androgenetic alopecia
	② Male participants diagnosed as Stage 2, 2A, or higher according to the Norwood–Hamilton classification
	③ Female participants diagnosed as Stage 1 or higher according to the Ludwig classification
	2. Participants who will not use specialized hair products or undergo hair treatments/manipulations during the study period
	3. Participants who will maintain the same hairstyle and hair color as at the initial visit throughout the study period
Exclusion criteria	1. Individuals with severe acute renal or cardiac diseases or other chronic conditions that may affect study results
	2. Individuals who are pregnant, breastfeeding, or planning to become pregnant within 6 months
	3. Individuals with psychiatric disorders or infectious skin diseases
	4. Individuals who have undergone surgical treatments for hair loss (e.g., hair transplant, scalp reduction)
	5. Individuals who have taken oral dutasteride or finasteride in the past 6 months
	6. Individuals who have taken medications or applied topical substance that may affect hair growth in the past month
	7. Individuals deemed unsuitable by the principal investigator

At each visit, eligible participants received a shampoo and hair tonic containing Asacurin100 (NJY Biotechnology, Republic of Korea), a natural plant‐based extract derived from *Acorus calamus* root, *Morus alba* bark, *Pinus thunbergii* leaf, *Sophora flavescens* root, *Cnidium officinale* root, and *Angelica gigas* root, extracted using low‐temperature vacuum extraction [9]. The quantity of the shampoo and the hair tonic was 500 and 100 mL, respectively. The whole formula of the shampoo and the hair tonic is presented in Table S1. Participants were instructed to apply the shampoo once daily and the hair tonic two to three times daily, spraying it ten times onto the affected areas. This treatment protocol was maintained for 6 months, with monthly clinic visits. The overall flow of the study is delineated and summarized in Table 2.

**TABLE 2:** Outline of the study.

	Screening	Treatment						
Visit	1	2	3	4	5	6	7	8
Informed consent	○							
Verification of inclusion/exclusion criteria	○							
Collection of medical and medication history	○							
Demographics	○							
Vital signs	○	○	○	○	○	○	○	○
Distribution of shampoo/tonic		○	○	○	○	○	○	○
Photography and questionnaire		○	○	○	○	○	○	○
Adverse event report			○	○	○	○	○	○

### 2.2 Data Collection

**FIGURE 1:**
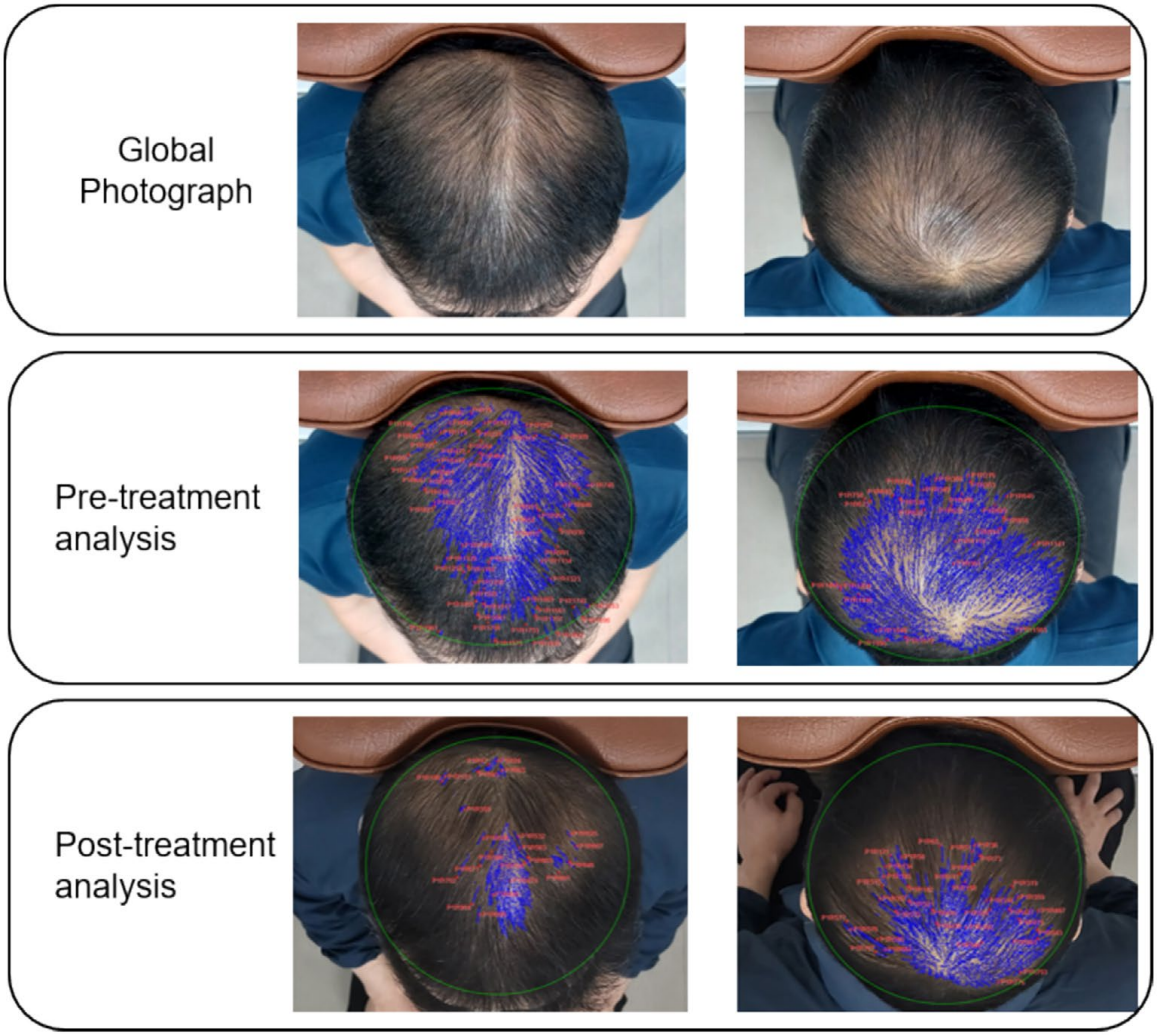
Example of a standardized global photograph and software analysis.

Baseline characteristics, including age, sex, height, weight, smoking history, and alcohol consumption, were recorded. At each visit, standardized global scalp photographs were taken using a digital camera (Galaxy Jump, SM‐A326K; Samsung Electronics Co., Korea) at a resolution of 4000 × 3000 pixels and a density of 720 dpi. To ensure consistency, all photographs were taken under the same conditions, capturing the frontal, anterior/mid, and crown views of the scalp. For accuracy, two sets of images were taken per participant: One with the participant facing forward and another after rotating 180°, while maintaining the same camera position (Figure 1). To ensure patient anonymity and confidentiality, only the scalp was included in the photographs, excluding any identifiable features. Consequently, additional consent for the use of the images was deemed unnecessary.

To evaluate hair and scalp conditions, a custom‐designed, 10‐item questionnaire was administered. The specific items of the questionnaire include: Excessive hair shedding, thinning of hair, lack of hair strength, scalp heat (redness), dandruff, scalp inflammation (pimples), scalp pain, excessive scalp oiliness, scalp dryness, and scalp itchiness (Table S2). Participants rated each item on a scale of 0 (no symptoms) to 10 (most severe symptoms). Additionally, the Patient Global Impression of Change (PGIC) questionnaire was administered at the final visit to evaluate overall hair loss improvement.

To enhance treatment adherence, the remaining quantities of shampoo and hair tonic were measured at each visit, and the usage ratio was calculated based on total product use per month. Participants with a usage ratio of < 60% were excluded from the study. Safety assessments were based on self‐reported adverse events.

### 2.3 Data Analysis

Baseline demographics were analyzed using descriptive statistics. Changes in the area of hair loss were evaluated using a paired *t*‐test. A multiple linear model was used to adjust for covariates, such as age, smoking history, alcohol consumption, and body mass index (BMI). Statistical significance was set at 0.05, and all analyses were conducted using SAS 9.4 (SAS Institute Inc., Cary, NC). Graphs were generated using R4.2.0 (https://cran_r‐project.org/). Continuous data were presented as means with 95% confidence intervals (CIs), whereas categorical data were reported as frequencies and percentages.

Photograph analysis was performed using Photoshop 2025 (ver. 25.9.0, Adobe, San Jose, CA) and Image‐Pro 10 (ver. 10.0.15, Media Cybernetics, Rockville, MD). Using Photoshop 2025, all areas except the scalp were removed without further adjustments, resulting in a scalp area of 1460 × 1090 pixels at 720 dpi. The resized images were then processed with Image‐Pro 10 to calculate the area of hair loss. The entire hair region was selected, and the threshold tool was employed to identify lighter areas indicative of hair loss. However, some lighter areas included white hair, which were manually excluded upon review to ensure accuracy (Figure 1). The primary outcome was the change in hair loss area rate between the initial and final photographs (final photo area − initial photo area/initial photo area × 100). To enhance reliability, the interclass correlation coefficient (ICC) was calculated for two sets of photographs, and analyses utilizing the mean value from the two sets of images were also conducted. All data analyses were conducted by a statistical expert and an image editing specialist who were not involved in the clinical study.

## 3 Results

**FIGURE 2:**
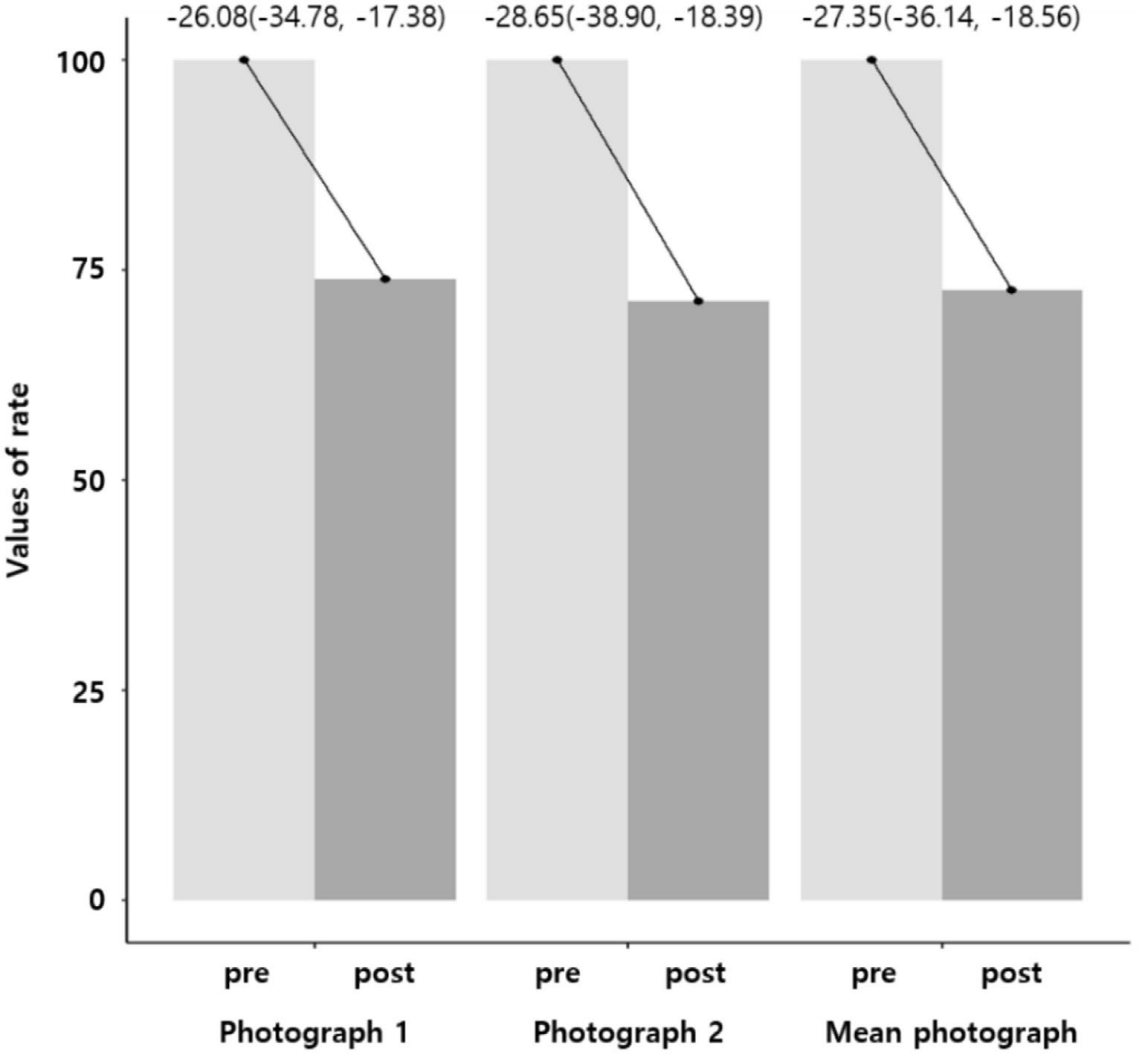
Change in hair loss area. Photographs 1 and 2 represent analyses of each of the two sets of photographs. The analysis was conducted using a multiple mixed model adjusted for age, height, weight, smoking, and alcohol consumption.

**FIGURE 3:**
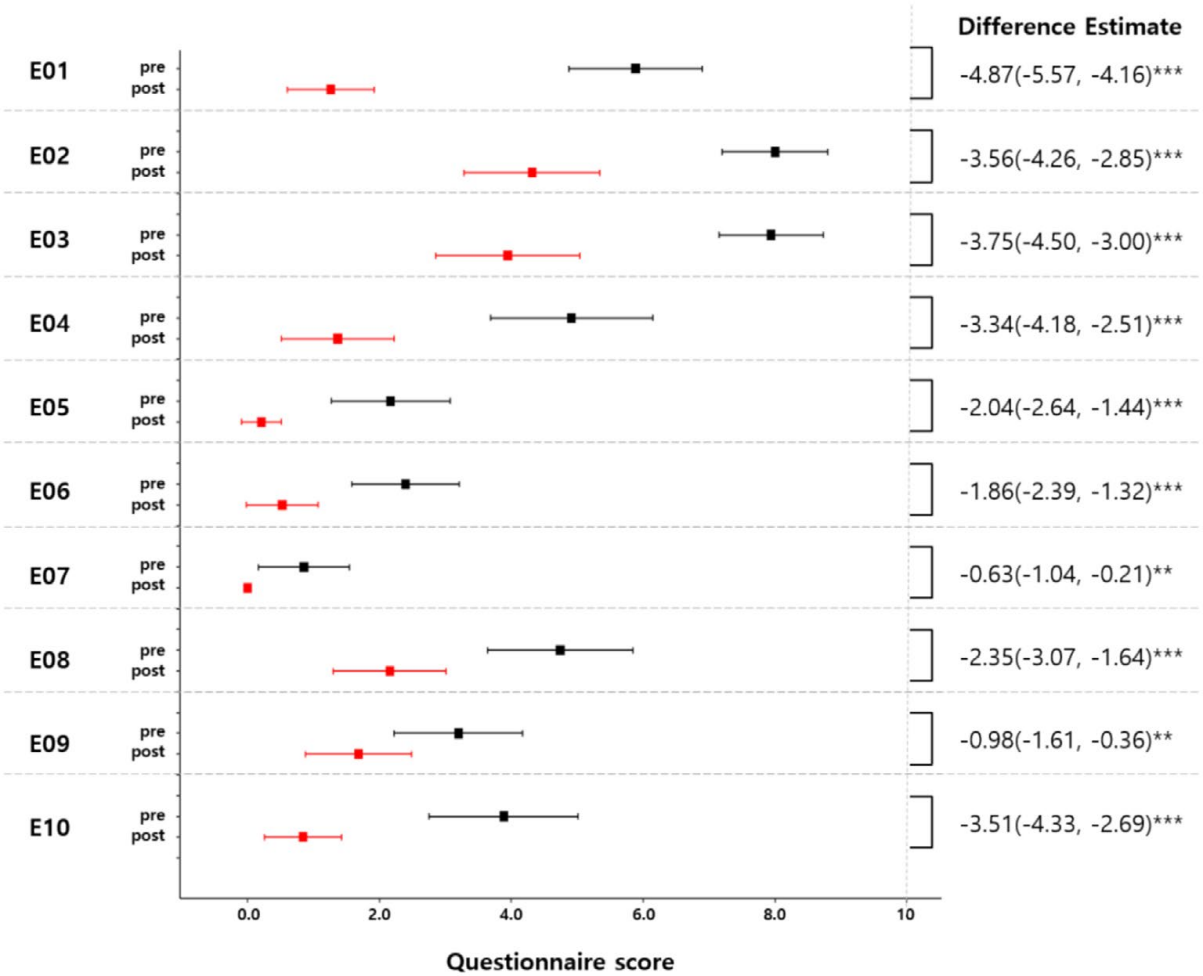
Changes in hair–scalp questionnaire scores. The difference estimate was derived from a multiple mixed model adjusted by age, weight, height, smoking, and alcohol consumption. **: *p* < 0.01, ***: *p* < 0.001.

Thirty‐five eligible participants were enrolled in the study, with no dropouts throughout the trial. All participants were male, with a mean age of 58.31 ± 5.57 years, and a mean BMI of 24.67 ± 2.74 kg/m^2^. Among them, 28.6% had a history of smoking, and 77.1% reported a history of alcohol consumption. A comparison of global photographs taken before and after the trial showed a reduction in the area of hair loss in both sets of photographs as well as the mean value of the photographs (−27.35 ± 26.31, *p* < 0.0001) (Figure 2). The interclass correlation coefficient (ICC) indicated moderate‐to‐good reliability (ICC: 0.776, 95% CI: 0.600–0.880, *p* < 0.0001). Complete pre‐ and posttreatment photographs of the participants are provided in the supplementary figure. All items in the scalp hair questionnaire demonstrated a significant improvement (Figure 3). In the PGIC assessment, 80% of the participants reported “much” or “very much” improvement. No serious adverse events were reported.

## 4 Discussion

AGA, the most prevalent hair loss disorder, is primarily influenced by genetic factors. Androgens, particularly DHT, play a critical role in AGA pathogenesis, with the 5‐alpha‐reductase enzyme acting as a key mediator [1]. Although genetic predisposition is widely recognized, recent literature suggests potential associations with environmental factors, metabolic syndrome, and microbial influences (microinflammation) [3, 4, 12]. Given these multifactorial contributors, a comprehensive management approach targeting both primary and secondary factors is recommended. Current treatment options include FDA‐approved topical minoxidil and oral finasteride, with limited use of dutasteride primarily in South Korea. Oral 5‐alpha‐reductase inhibitors, such as finasteride and dutasteride, are commonly prescribed as first‐line treatments for AGA. Numerous studies have demonstrated the efficacy of these drugs, with Korean research indicating a favorable safety profile [13]. Finasteride, administered at a dose of 1 mg daily, is generally well tolerated, with side effects typically resolving upon discontinuation. However, there is ongoing debate regarding its safety, as some individuals experience persistent side effects for at least 3 months postcessation [1, 12]. For topical treatment, the FDA has approved 2% and 5% minoxidil formulations. While effective, its efficacy was lesser than oral 5‐alpha‐reductase inhibitors [14] and itching of scalp, erythema is a commonly reported side effect [15]. Despite the availability of these treatments, their efficacy can be suboptimal, and side effects often lead to decreased patient adherence [1, 4, 5]. Botanically derived therapies are gaining popularity owing to their cost‐effectiveness and lower risk of adverse effects. Various plant extracts have been shown to enhance the survival and proliferation of dermal papilla cells, stimulate hair follicle cell growth, and promote hair growth in both in vitro and in vivo studies. These effects are linked to improved cell survival and proliferation, upregulation of growth factors, and the mitigation of oxidative stress and inflammatory response, along with the downregulation of male hormones and their receptors [16]. Among natural plant‐derived treatments, *Serona repens* (saw palmetto) and rosemary oil have received significant research attention. *Serona repens*, derived from the berries of the American dwarf palm tree, reduces DHT levels by inhibiting 5‐alpha‐reductase. Several clinical trials have demonstrated its efficacy, with some studies indicating superiority over placebo [17]. Rosemary oil is recognized for its antioxidant, antimicrobial, and anti‐inflammatory properties and has shown effects comparable to topical minoxidil [17]. Additionally, curcuma aeruginosa, another botanical 5‐alpha‐reductase inhibitor, has demonstrated similar efficacy to topical minoxidil [5]. However, many trials lack robust design, appropriate outcomes, and sufficient sample sizes. The inconsistent findings and a lack of randomized controlled trials limit the inclusion of natural products in evidence‐based guidelines, further necessitating further research to establish the definitive efficacy of these botanical treatments.

The natural plant extract blend used in the study has demonstrated anti‐inflammatory effects in atopic dermatitis and the upregulation of hair growth‐promoting genes in animal models [9]. Herbs included in the blend, *Acorus calamus* , *Morus alba, Glycyrrhiza, Pinus thunbergii*, *Sophora flavescens* , *Cnidium officinale*, and *Angelica* gigas, have been traditionally and empirically used in South Korea for hair care. Recent studies suggest that inflammation is a feature of AGA, with cytokines potentially playing a detrimental role by affecting hair growth. Microbial overcolonization of the pilosebaceous unit is believed to be a primary contributor to microinflammation [4, 15]. The potential mechanisms of action for this natural plant extract blend include the anti‐inflammatory, antibacterial, and antifungal properties of *Acorus calamus* , *Pinus thunbergii, Cnidium officinale*, and *Angelica* gigas [18, 19, 20, 21]. Additionally, *Morus alba* and *Sophora flavescens* may enhance the hair growth environment by influencing the growth factors of derma papilla cells [22, 23]. Furthermore, *Sophora flavescens* has been shown to inhibit the catalytic activity of steroid 5‐alpha‐reductase type II, contributing to its potential efficacy in AGA management [16]. This study evaluated the efficacy of a botanical extract–infused shampoo and hair tonic in patients with AGA, and the results showed a significant reduction in hair loss, improvement in scalp symptoms, and high treatment tolerance. Unlike conventional treatments for AGA, which typically rely on a single active agent, the botanical extracts contain multiple bioactive compounds. Considering the multifactorial nature of AGA and the increasing use of combination therapies, a multimodal approach could be advantageous. Plant‐derived compounds may serve as effective standalone treatments or adjuncts to conventional therapies, offering patient‐relevant benefits.

A key strength of this study is its objective evaluation of global photographs for AGA assessment using computer software and independent evaluators. Compared to other major dermatological conditions, hair loss disorders lack definitive diagnostic and severity assessment methods. The paucity of evidence‐based guidelines and assessment by individuals represents potential for human error. To address the underlying problem of global photograph assessment, we utilized computer software and independent evaluators to minimize the bias. Additionally, given the growing emphasis on patient‐centered medicine, this study incorporated a custom‐designed questionnaire and PGIC scale to capture patients' subjective experiences and treatment perceptions [24].

Despite these promising findings, several limitations should be acknowledged. First, as a single‐arm study, the independent effects of the botanical extract–infused shampoo and hair tonic could not be accurately assessed. Without a parallel control group and blinding process, there could be biases in interpreting the results. Nevertheless, researchers who did not directly participate in the trial analyzed the patients' data to minimize bias. Moreover, all participants were male and recruited from a single clinic in Korea, limiting the generalizability of the findings to other populations. These factors underscore the need for larger randomized controlled trials. Additionally, basic research is needed to delve into the specific mechanism of the compound. Second, the photo analysis method and custom‐designed questionnaire lacked formal content validity assessment. While efforts were exerted to ensure objectivity, manual adjustment of brightness thresholds and exclusion of gray hair areas could introduce bias. Future research should focus on developing more objective assessment metrics, such as applying machine learning techniques to large‐scale scalp imagery, to enhance reliability and reproducibility. Third, while it is necessary to consider various parameters, this study only assessed the area of hair loss and subjective symptoms. Regional features of the scalp, such as focal atrichia, may be overlooked in global scalp photography. Improvements in AGA should also be evaluated in terms of hair density and thickness, as common symptoms include increased shedding and thinning. Automated digital photographic systems such as TrichoScan offer objective and standardized assessments of hair density and thickness. Nonetheless, TrichoScan requires shaving and dyeing, which could impact study compliance, requires specialized equipment and software, and it presents limitations related to the analysis area, degree of hair loss, and potential errors [25, 26]. Additionally, global photographic assessment is deemed the most effective method for evaluating hair growth, as it eliminates subjective perceptions from both patients and investigators [27]. Given that this study is preliminary, aimed at exploring feasibility and data collection, global photography was considered the most practical method. Future research should consider the diverse dimensions of AGA, encompassing a wide range of signs and symptoms, and should aim to optimize software analysis.

## 5 Conclusion

In summary, the findings of this study suggest that a botanical extract–infused shampoo and hair tonic may effectively reduce hair loss and improve scalp conditions in patients with AGA. The plant‐derived compounds show potential as an alternative or adjunct to conventional therapies.

## Ethics Statement

This study was approved by the Public Institutional Review Board of the Ministry of Health and Welfare (Approval No. P01‐202307‐01‐010). This study was conducted in accordance with the principles of the Declaration of Helsinki.

## Conflicts of Interest

The authors declare no conflicts of interest.

## Supporting information


**Data S1.** Supporting Information.

## Data Availability

The data that support the findings of this study are available on request from the corresponding author. The data are not publicly available due to privacy or ethical restrictions.
