# Supplementary material for: Botanical Extract–Infused Shampoo and Hair Tonic for Hair Loss in Androgenetic Alopecia: A TREND‐Compliant, Prospective Single‐Arm Preexperimental Study
Source: J Cosmet Dermatol. 2025 Jun 4;24(6):e70273. doi: 10.1111/jocd.70273 (PMC12137762; doi:10.1111/jocd.70273)
Supplement: Supplementary file 1 — Data S1. Supporting Information. [file JOCD-24-e70273-s001.docx]

**Supplementary Figure. Pre post participant global scalp photograph**

**Participant 1.**


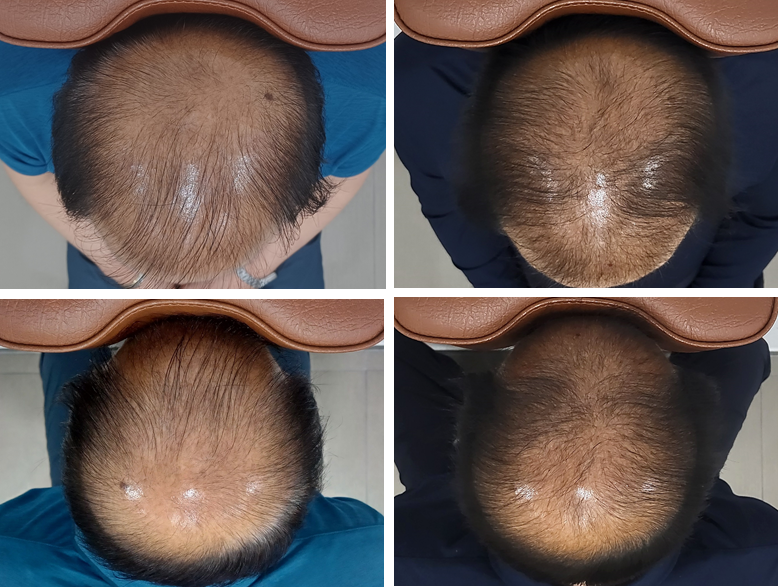


Left: pre treatment, Right: post treatment

**Participant 2.**


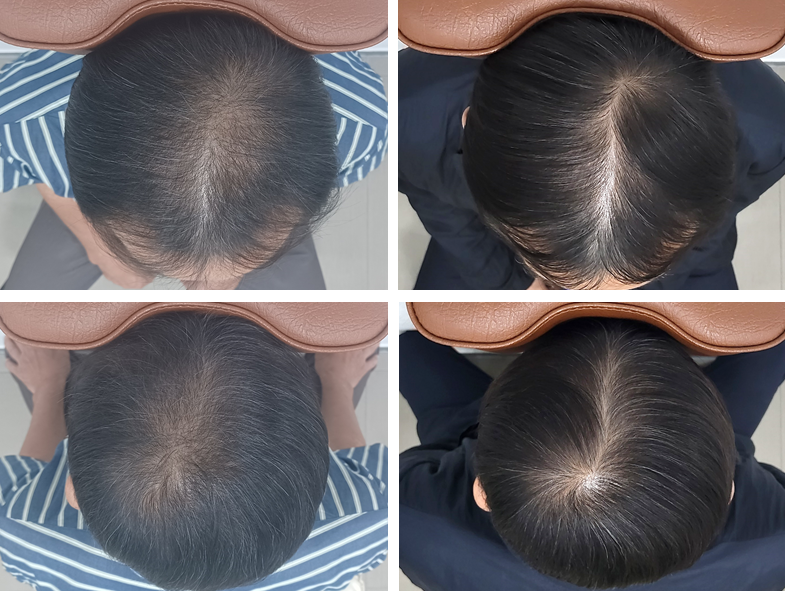


Left: pre treatment, Right: post treatment

**Participant 3.**

**
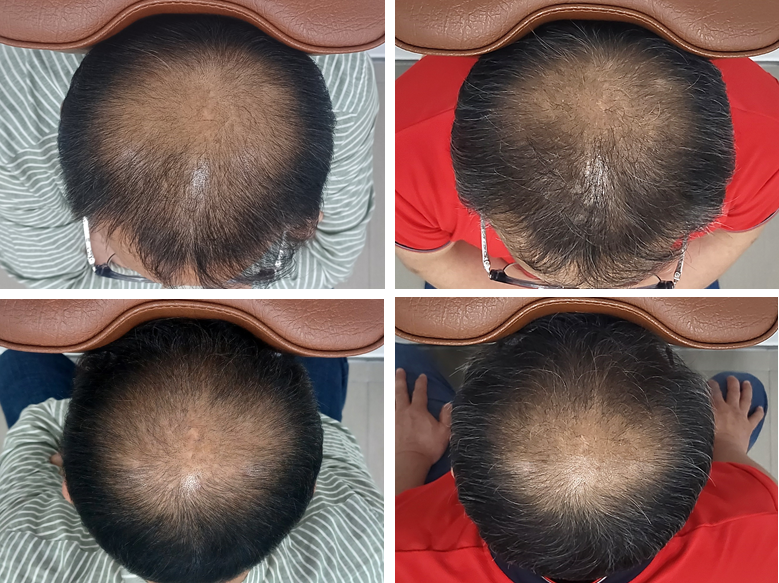
**

Left: pre treatment, Right: post treatment

**Participant 4.**

**
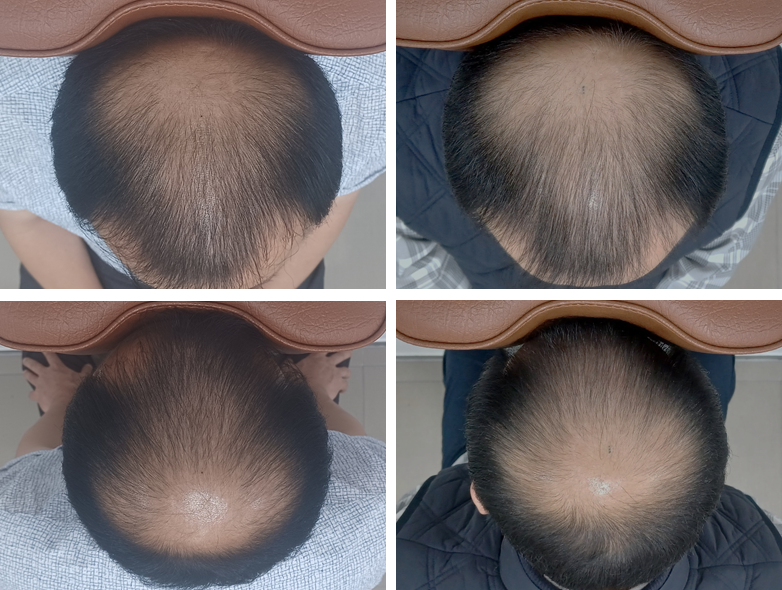
**

Left: pre treatment, Right: post treatment

**Participant 5.**

**
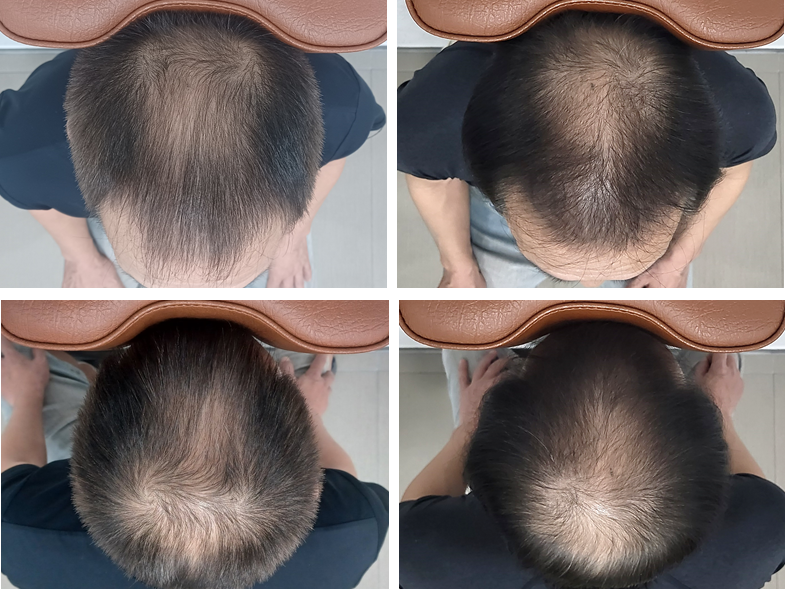
**

Left: pre treatment, Right: post treatment

**Participant 6.**

**
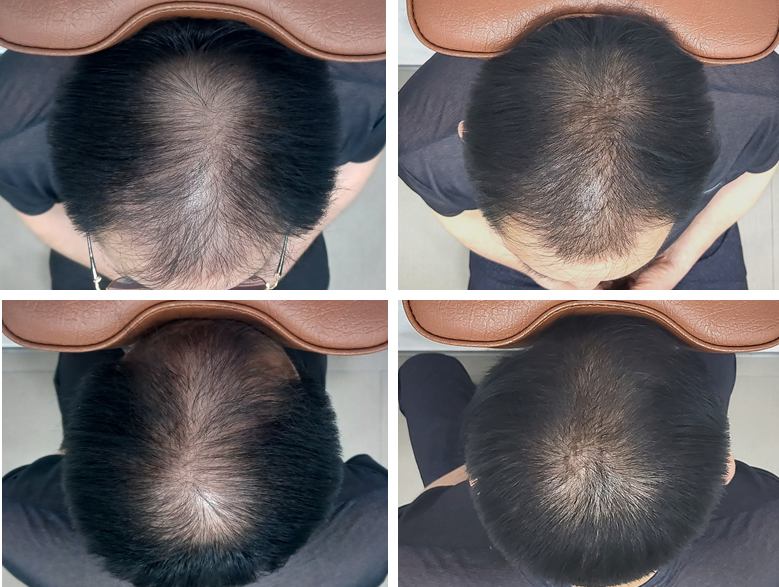
**

Left: pre treatment, Right: post treatment

**Participant 7.**

**
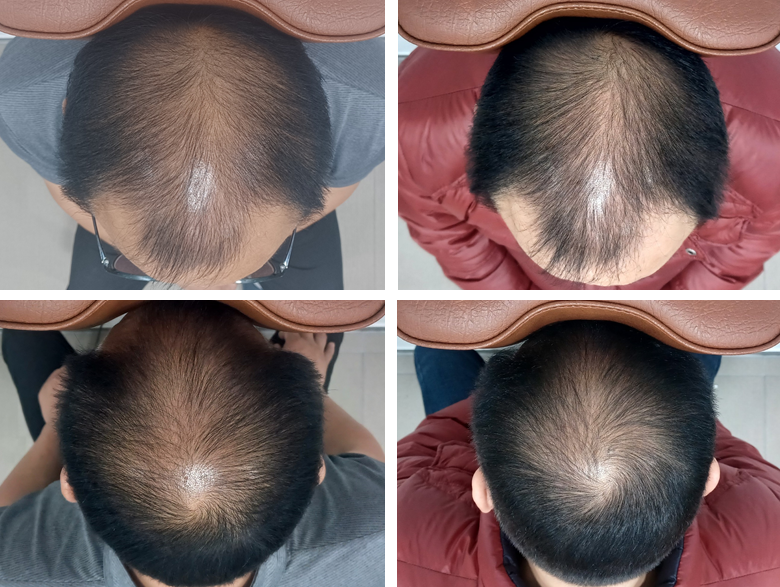
**

Left: pre treatment, Right: post treatment

**Participant 8.**

**
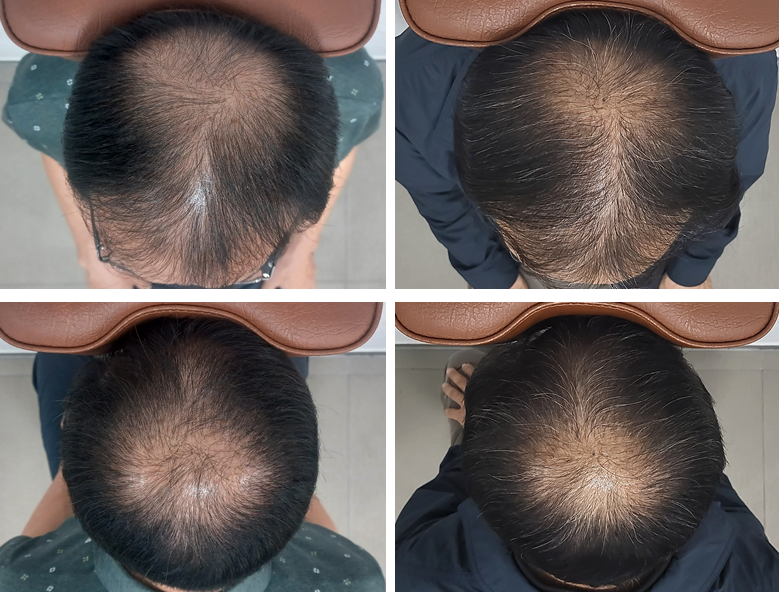
**

Left: pre treatment, Right: post treatment

**Participant 9.**

**
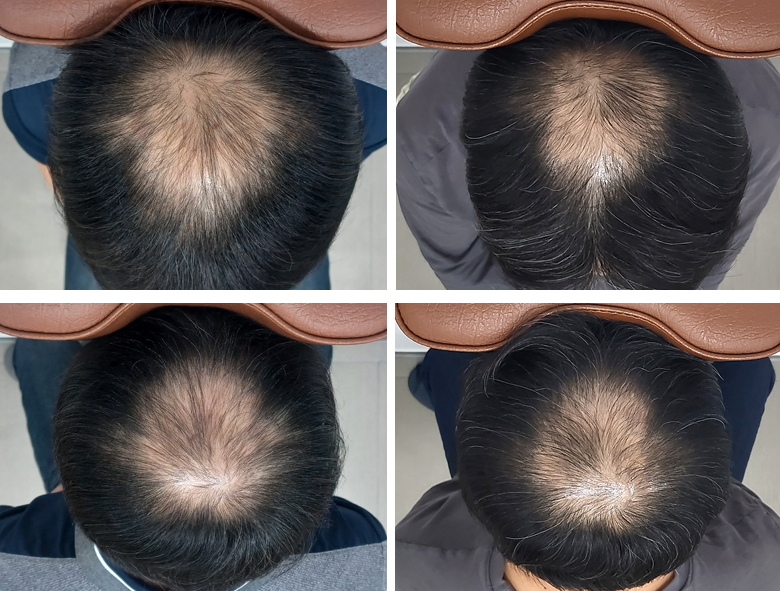
**

Left: pre treatment, Right: post treatment

**Participant 10.**

**
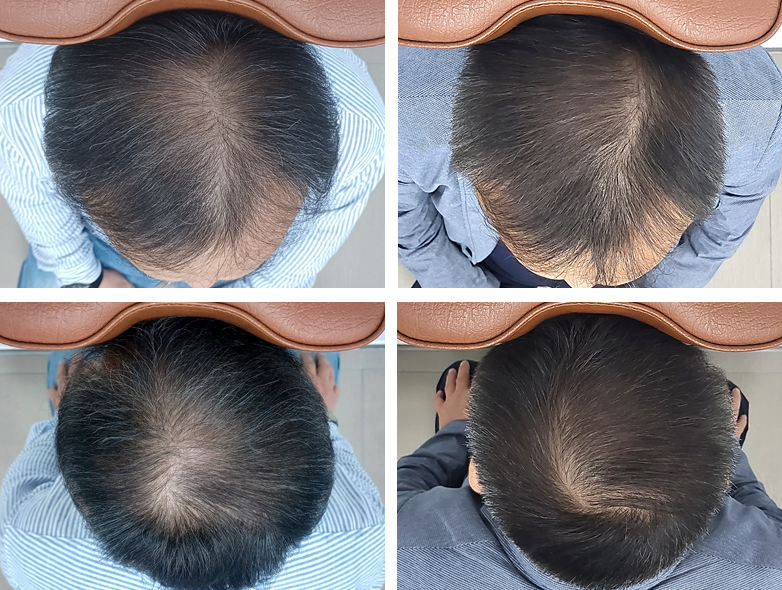
**

Left: pre treatment, Right: post treatment

**Participant 11.**

**
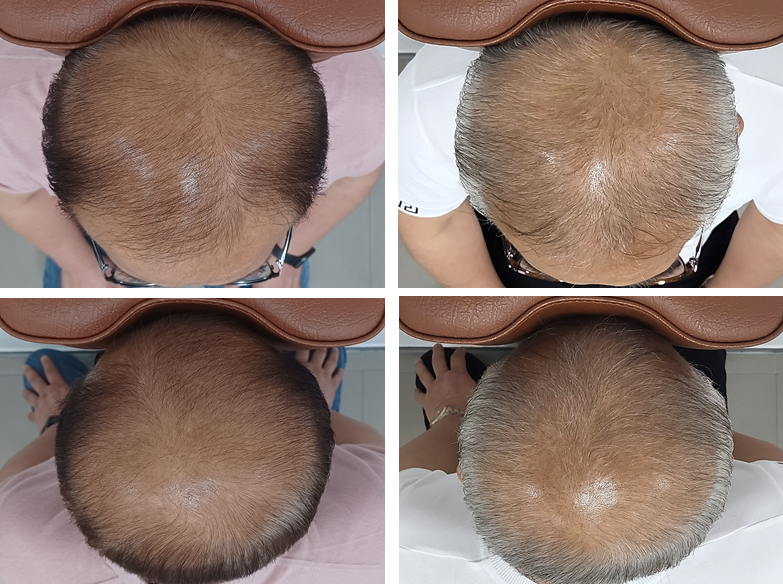
**

Left: pre treatment, Right: post treatment

**Participant 12.**

**
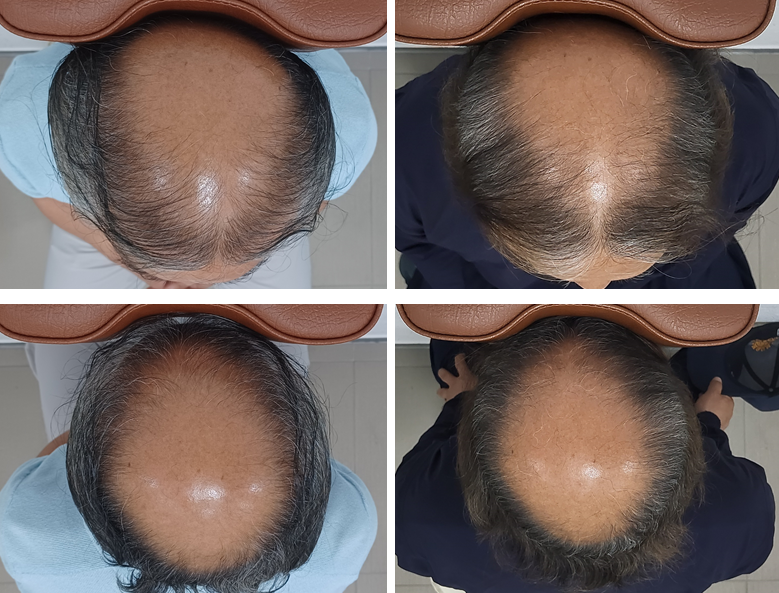
**

Left: pre treatment, Right: post treatment

**Participant 13.**

**
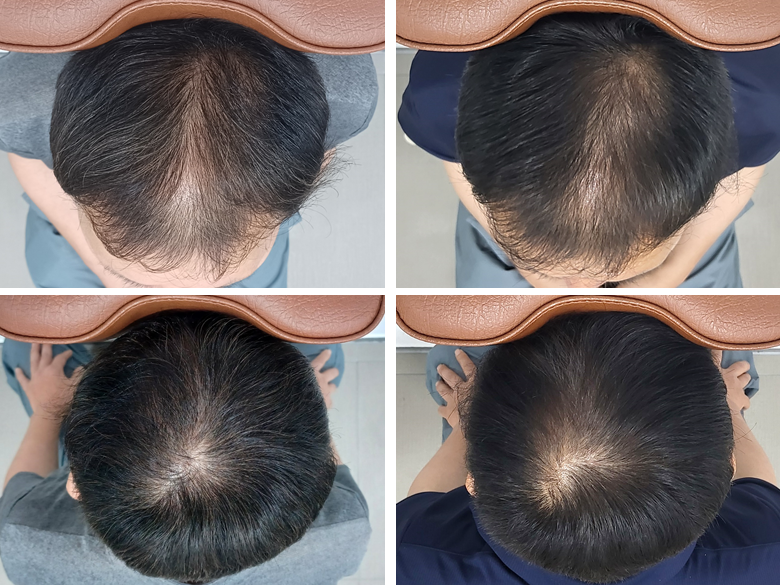
**

Left: pre treatment, Right: post treatment

**Participant 14.**

**
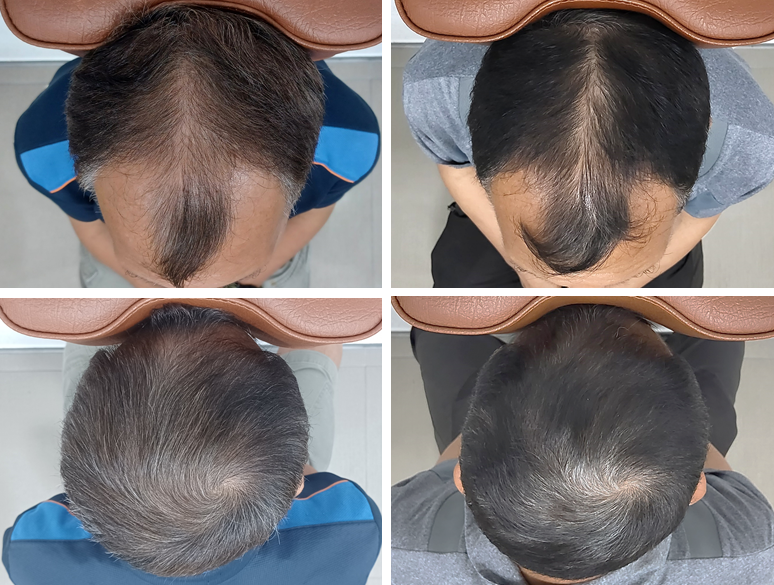
**

Left: pre treatment, Right: post treatment

**Participant 15.**

**
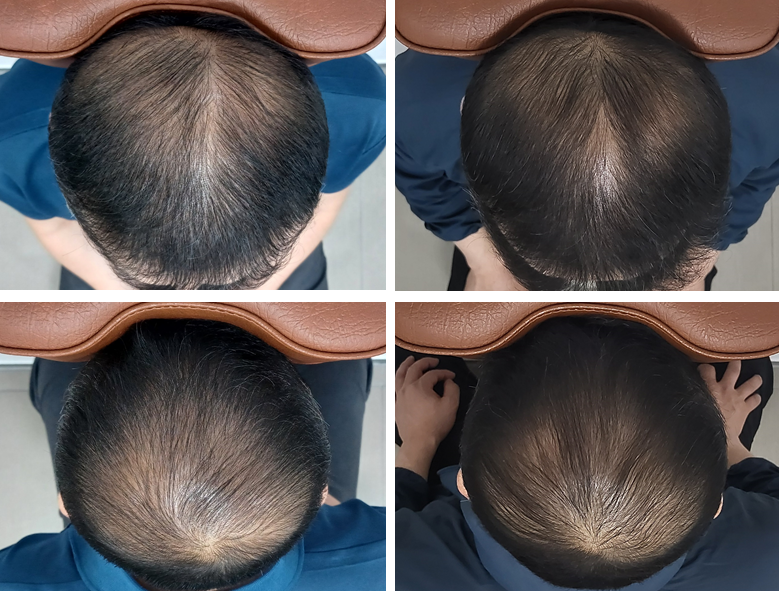
**

Left: pre treatment, Right: post treatment

**Participant 16.**

**
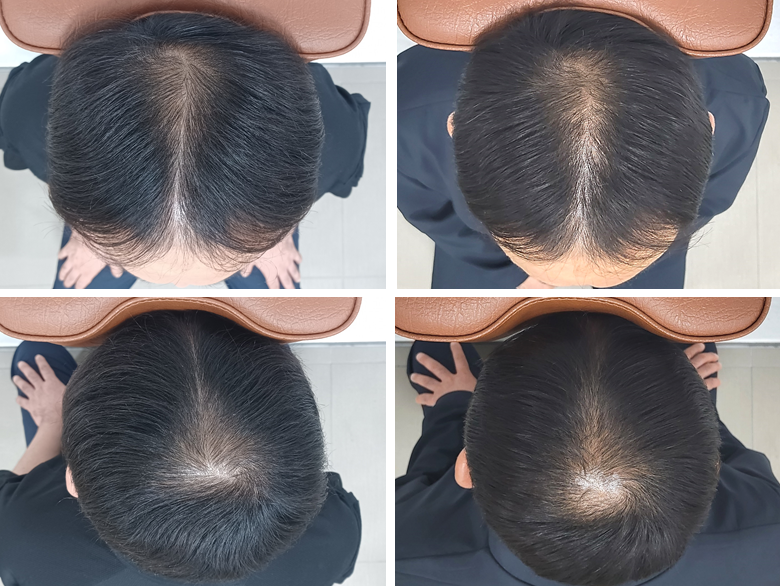
**

Left: pre treatment, Right: post treatment

**Participant 17.**

**
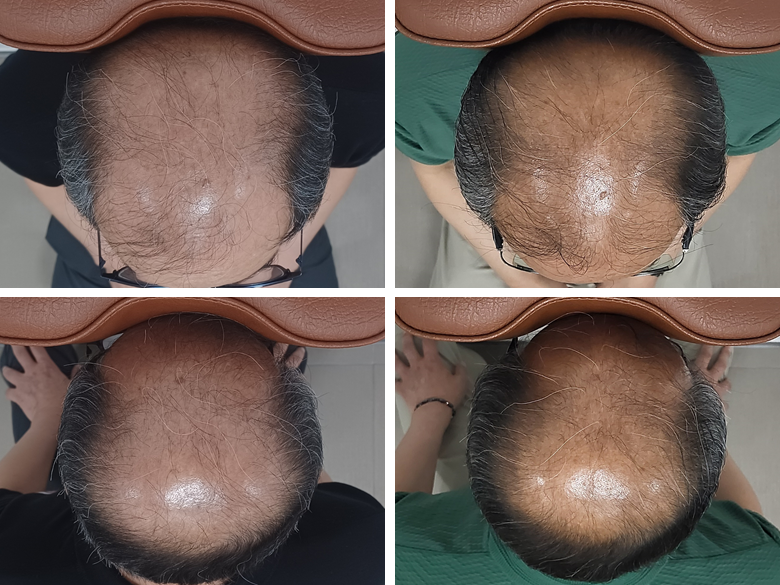
**

Left: pre treatment, Right: post treatment

**Participant 18.**

**
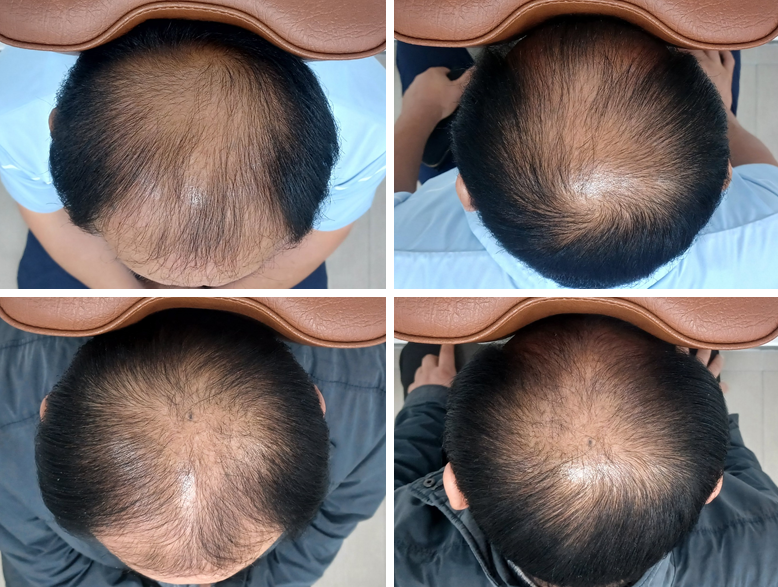
**

Left: pre treatment, Right: post treatment

**Participant 19.**

**
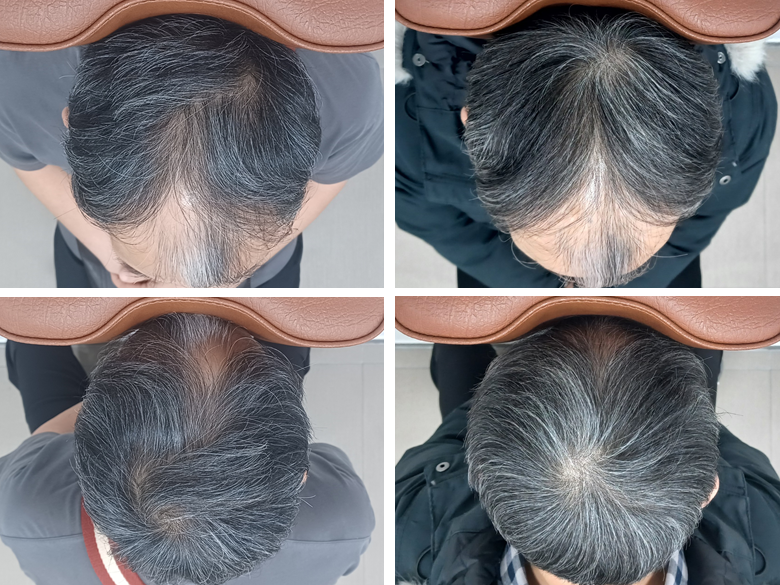
**

Left: pre treatment, Right: post treatment

**Participant 20.**

**
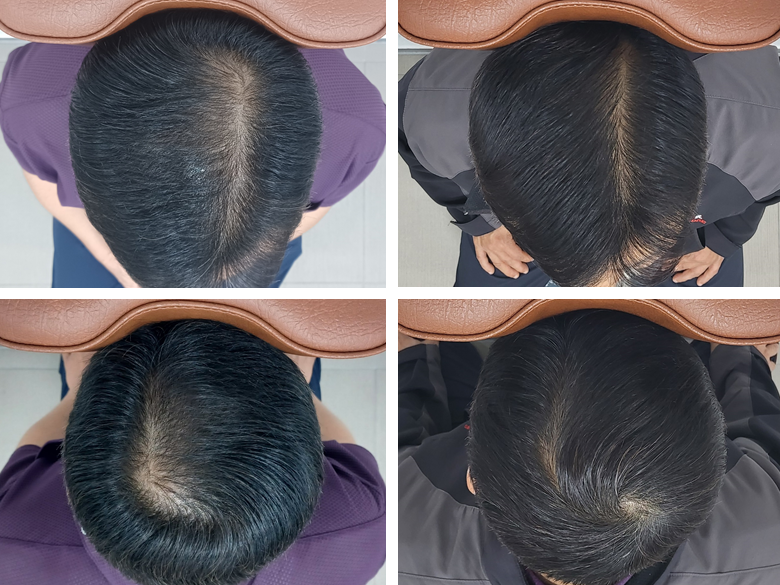
**

Left: pre treatment, Right: post treatment

**Participant 21.**

**
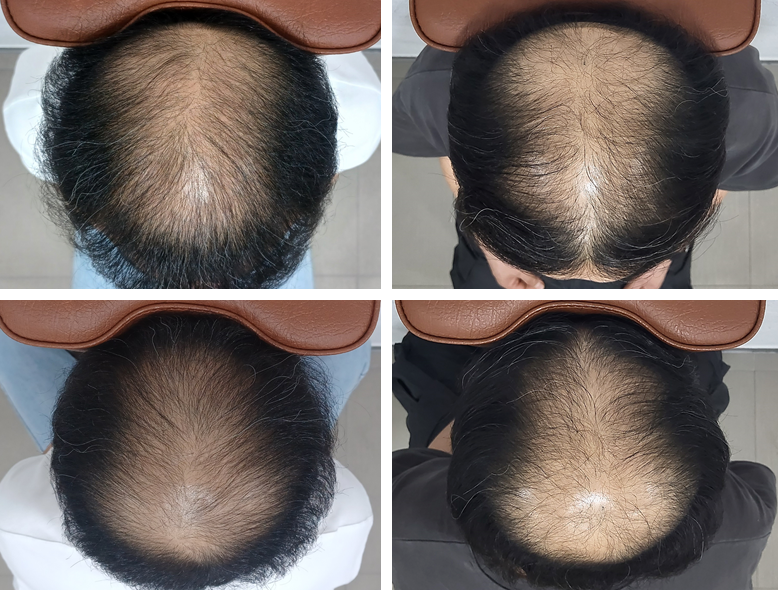
**

Left: pre treatment, Right: post treatment

**Participant 22.**

**
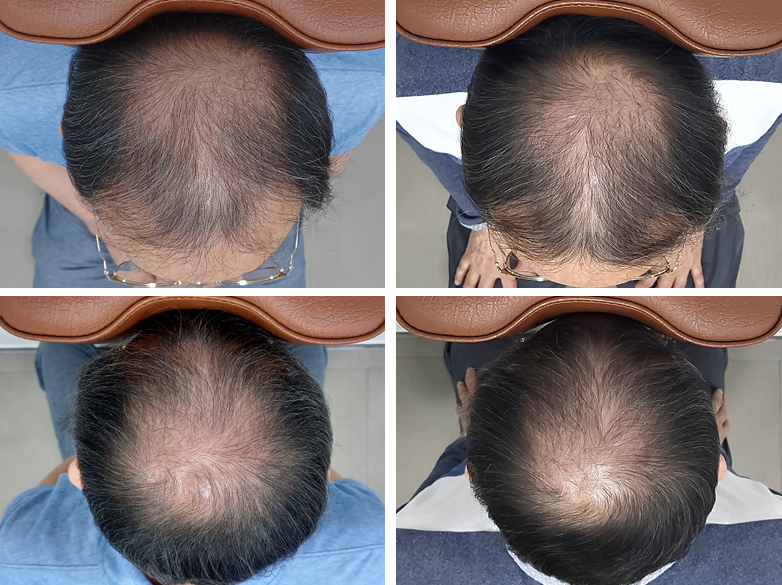
**

Left: pre treatment, Right: post treatment

**Participant 23.**

**
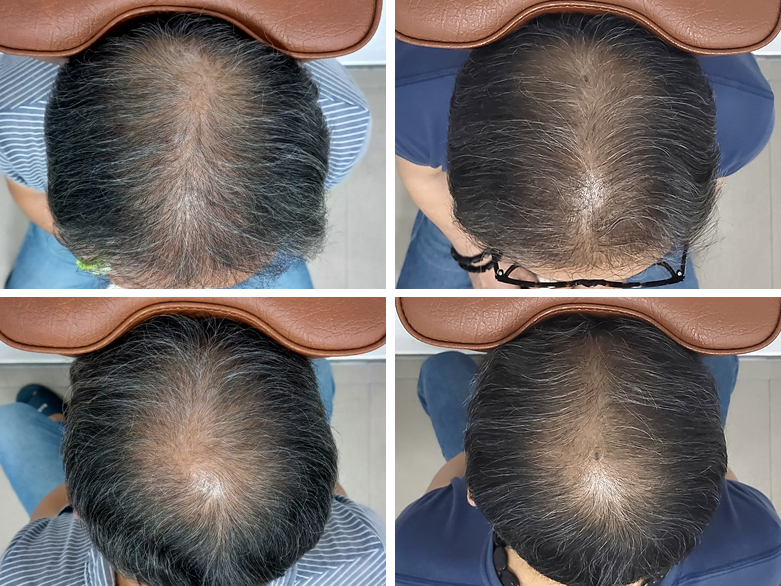
**

Left: pre treatment, Right: post treatment

**Participant 24.**

**
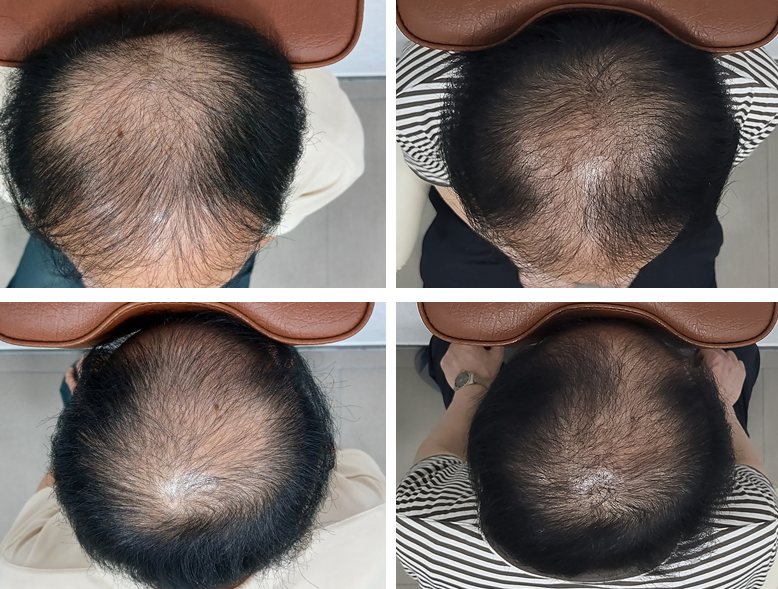
**

Left: pre treatment, Right: post treatment

**Participant 25.**

**
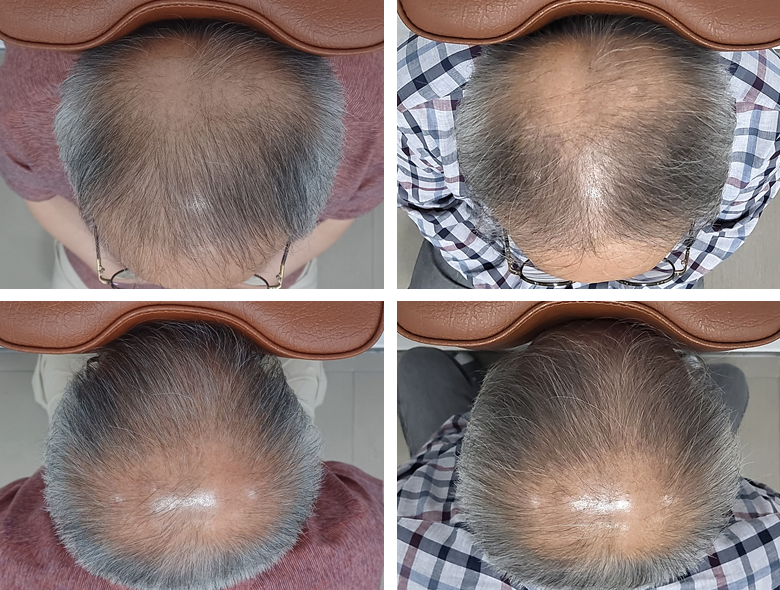
**

Left: pre treatment, Right: post treatment

**Participant 26.**

**
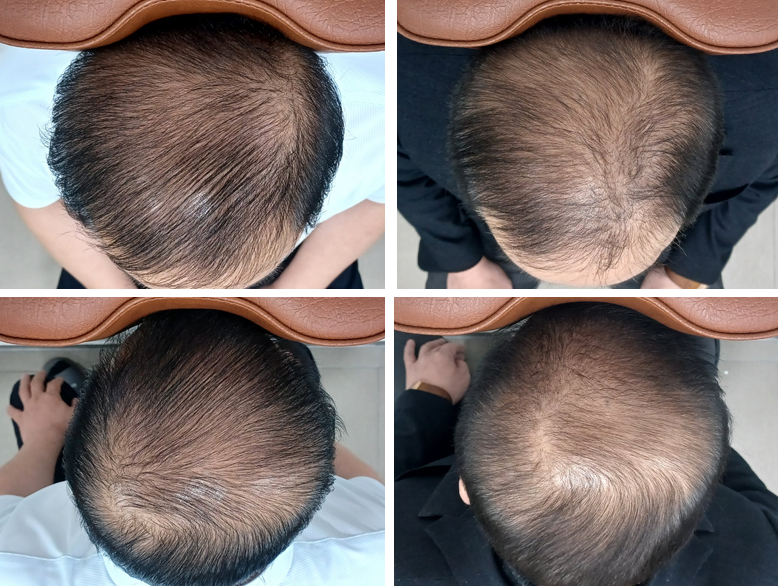
**

Left: pre treatment, Right: post treatment

**Participant 27.**

**
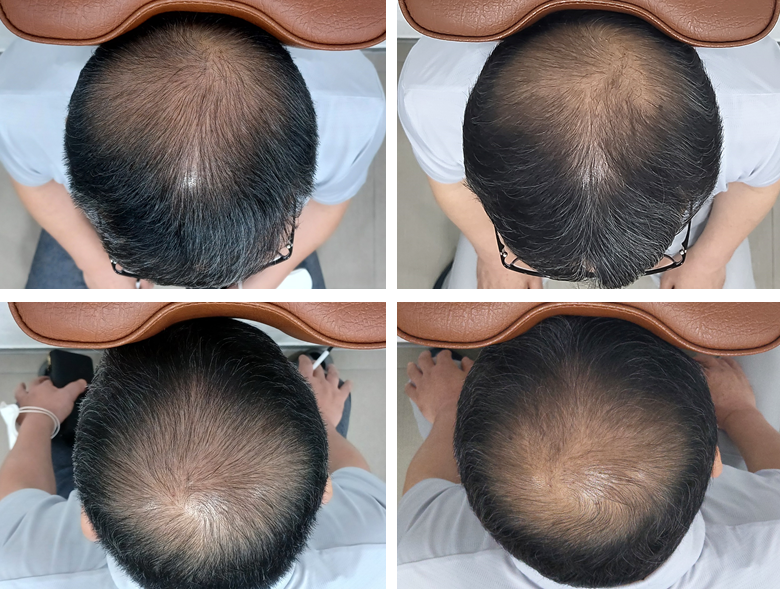
**

Left: pre treatment, Right: post treatment

**Participant 28.**

**
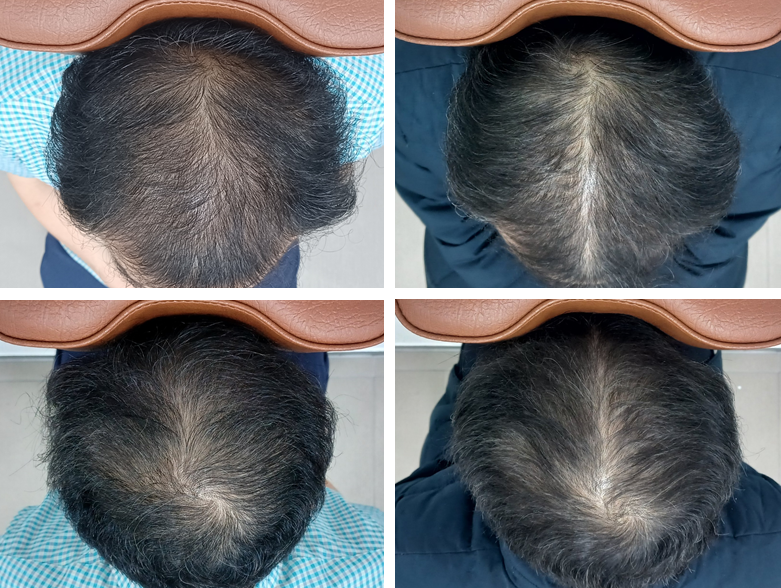
**

Left: pre treatment, Right: post treatment

**Participant 29.**

**
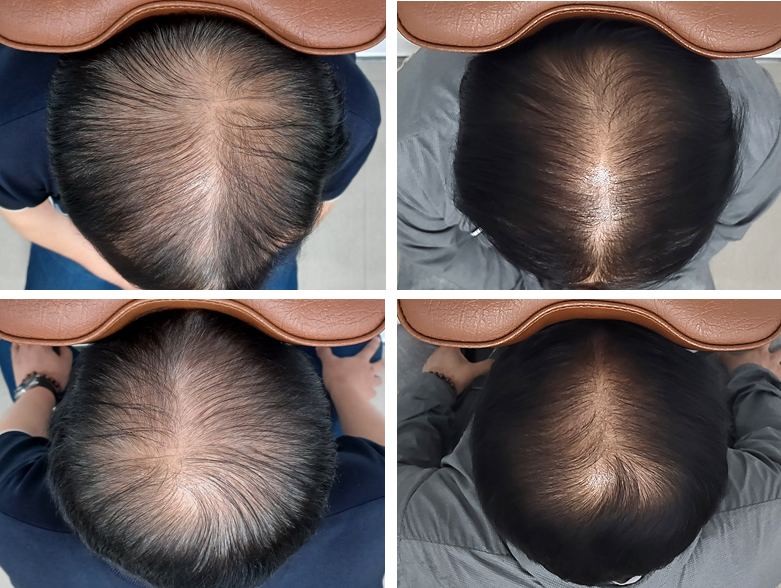
**

Left: pre treatment, Right: post treatment

**Participant 30.**

**
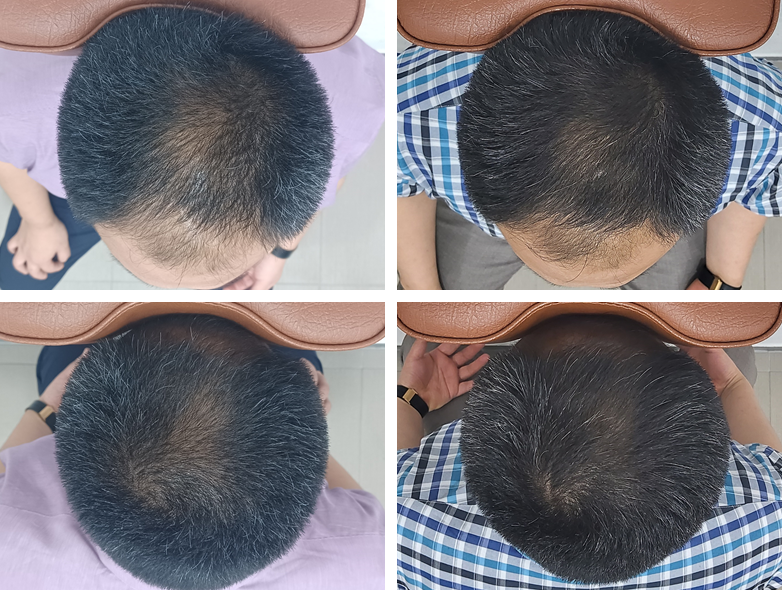
**

Left: pre treatment, Right: post treatment

**Participant 31.**

**
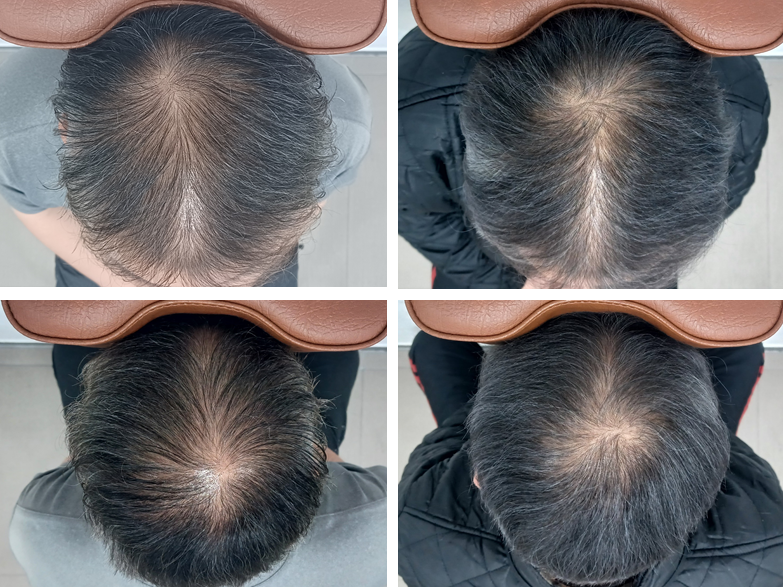
**

Left: pre treatment, Right: post treatment

**Participant 32.**

**
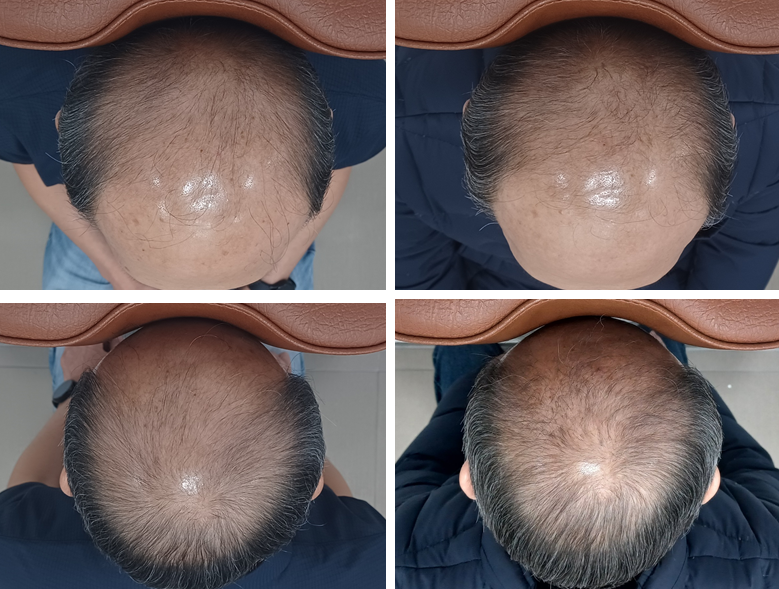
**

Left: pre treatment, Right: post treatment

**Participant 33.**

**
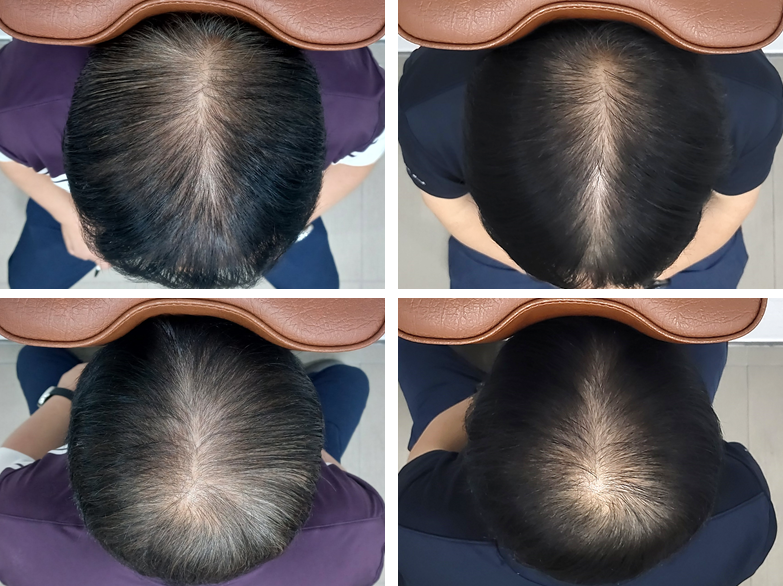
**

Left: pre treatment, Right: post treatment

**Participant 34.**

**
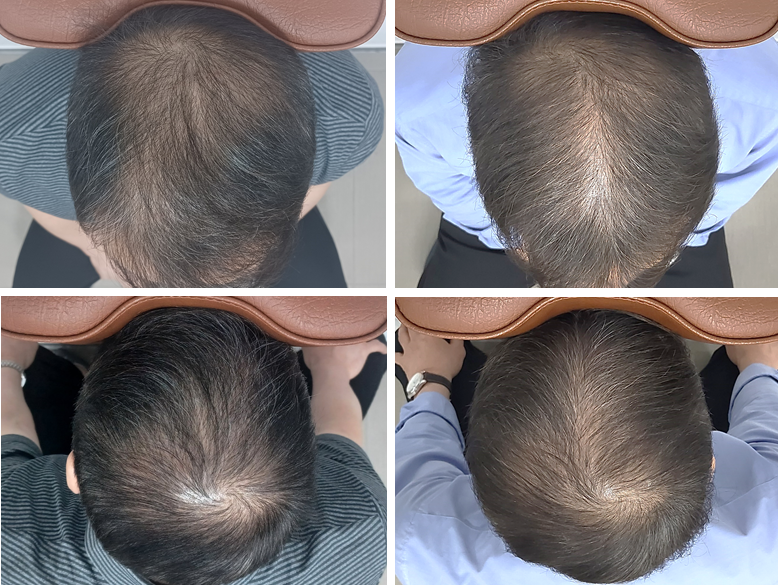
**

Left: pre treatment, Right: post treatment

**Participant 35.**

**
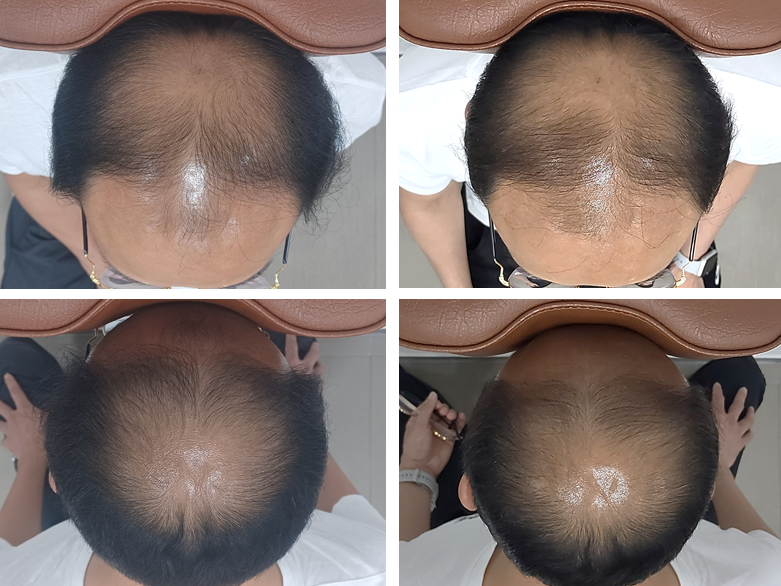
**

Left: pre treatment, Right: post treatment

**Supplementary Table 1. Composition of shampoo and hair tonic**

| Shampoo | Sodium C14-16 Olefin sulfonate, Disodium Laureth sulfosuccinate, Glycerin, Cocamide MIPA, Coco-glucoside, Lauryl Betaine, Polyquaternium-10, Polyquaternium-11, Hexadecene, Tetradecene, Sodium sulfate, Acorus Calamus root, Morus Alba bark, Pinus Thunbergii leaf, Sophora Flavescens root, Cnidium Officinale root, Angelica Gigas root, Portulaca Oleracea, Centella Asiatica, Houttuynia Cordata, Acorus Gramineus, Saposhnikovia Divaricata root, Astragalus Membranaceus root, Eriobotrya Japonica leaf, Saururus Chinensis, Thuja Orientalis, Artemisia Princeps leaf, Butylene glycol, Hydroxyacetophenone, Laurylpyridinium chloride, Caramel, Sodium chloride, Mentol, Salicylic acid, Panthenol, Citric acid, Sodium bicarbonate, Disodium EDTA |
| --- | --- |
| Hair tonic | Ethanol, Acorus Calamus root, Morus Alba bark, Pinus Thunbergii leaf, Sophora Flavescens root, Cnidium Officinale root, Angelica Gigas root, Portulaca Oleracea, Centella Asiatica, Houttuynia Cordata, Acorus Gramineus, Saposhnikovia Divaricata root, Astragalus Membranaceus root, Eriobotrya Japonica leaf, Saururus Chinensis, Thuja Orientalis, Artemisia Princeps leaf, Polyglyceryl-10 Stearate, Polyglyceryl-10 Oleate, Sodium chloride, Mentol, Salicylic acid, Panthenol, Citric acid, Caprylyl glycol, Pentylene glycol |

**Supplementary Table2. Hair and scalp questionnaire**

| Record the severity of symptoms for each item using the numerical rating scale from 0 to 10.  (0: no symptoms, 5: moderate, 10: most severe) | |
| --- | --- |
| Hair shedding |  |
| Hair thinning |  |
| Lack of hair strength |  |
| Scalp heat (redness) |  |
| Dandruff |  |
| Scalp inflammation (pimples) |  |
| Scalp pain |  |
| Oily scalp |  |
| Scalp dryness |  |
| Scalp itching |  |
